# Nocardia keratitis: amikacin nonsusceptibility, risk factors, and treatment outcomes

**DOI:** 10.1186/s12348-022-00287-1

**Published:** 2022-03-05

**Authors:** Ethan Adre, Jorge Maestre-Mesa, Heather Durkee, Alejandro Arboleda, Harry Flynn, Guillermo Amescua, Jean-Marie Parel, Darlene Miller

**Affiliations:** 1 University of Miami Miller School of Medicine, Miami, FL USA; 2 Ophthalmic Biophysics Center, Bascom Palmer Eye Institute, Department of Ophthalmology University of Miami Miller School of Medicine, Miami, FL USA; 3 Ocular Microbiology Laboratory, Anne Bates Leach Eye Center, Bascom Palmer Eye Institute, University of Miami Miller School of Medicine, McKnight Research Pavilions, Rm 103A,1638 NW 10th Avenue, Miami, FL 33136 USA; 4 Department of Ophthalmology, Bascom Palmer Eye Institute, University of Miami Miller School of Medicine, Miami, FL USA

**Keywords:** *Nocardia* keratitis, Antibiotic resistance, Contact-lens keratitis

## Abstract

**Purpose:**

To report the increasing trends in *Nocardia* keratitis species diversity and in vitro antibiotic susceptibility, to demonstrate contact lens wear as a risk factor, and to report visual acuity outcomes after treatment.

**Methods:**

A retrospective clinical case series was performed at a single academic referral center which identified 26 patients with culture-confirmed *Nocardia* keratitis between 2014 and 2021. A combination of conventional microbiology and molecular techniques were used to identify isolates. Antibiotic susceptibilities were determined using both commercial and in-house laboratory methods. Microbiology and electronic medical records were used to characterize patients’ clinical profiles.

**Results:**

Patients’ median age was 32.5 years with a 2:1 male to female ratio. Eighty-four percent (*n* = 21/25) of patients were diagnosed within two weeks of symptom onset. *Nocardia amikacinitolerans (n* = 11/26*)* was the most recovered *Nocardia* isolate among study patients*.* Sixty-four percent (*n* = 16/25) of all isolates, including all 11 *N. amikacinitolerans* isolates, were resistant to amikacin. All isolates were susceptible to trimethoprim sulfamethoxazole. Contact lens wear was the leading identified risk factor (*n* = 23/26) in this population. Median time to resolution was 44 days (*n* = 23, range: 3–190 days). Seventy-one percent of patients (*n* = 15/21) had a final visual acuity of 20/40 or better.

**Conclusion:**

Amikacin resistant *Nocardia* isolates were the majority in the current study. Trimethoprim sulfamethoxazole may be the preferred alternative antibiotic treatment based on in vitro susceptibilities. Contact lens wear was the major risk factor for *Nocardia* keratitis in South Florida. Overall visual acuity treatment outcomes of patients were favorable.

## Background

*Nocardia* are a heterogenous group of aerobic, branching, gram positive, weakly acid-fast bacteria commonly found in dust, decaying vegetable matter, and aquatic environments [1]. Ocular nocardiosis most often presents as keratitis [2, 3]. *Nocardia* keratitis is a rare, chronic, debilitating cause of keratitis historically associated with trauma [2–6]. Global prevalence is below 2% [2, 5]. It is difficult to diagnose and treat due to a combination of diverse species’ presentations and species-specific response to commonly used topical antibiotics.

Topical amikacin is the current standard of care for medical management of *Nocardia* keratitis [5, 7]. However, isolates are increasingly diverse and may differ by geography, patient population, and antimicrobial susceptibility [1, 2, 5]. Data on clinical presentation, risk factors, species diversity, and medical management have been reported predominantly for patient populations outside the United States. The purpose of the current study is to characterize and report *Nocardia* keratitis species diversity and in vitro antibiotic susceptibility, to identify contact lens wear as a risk factor among *Nocardia* keratitis patients, and to report visual acuity outcomes after treatment.

## Methods

The current study is a retrospective, single center, clinical case series. Institutional Review Board (IRB) approval was obtained from the University of Miami Miller School of Medicine Sciences Subcommittee for the Protection of Human Subjects and the research adhered to the Tenets of the Declaration of Helsinki (IRB Protocol Study ID #20070960). Clinical data was collected and analyzed for 26 patients presenting with *Nocardia* keratitis between January 2014 and September 2021. Extracted data included patient demographics, risk factors, days from symptom onset to presentation, presenting best corrected visual acuity (BCVA), days to resolution, BCVA at last follow-up, and topical steroid use.

A combination of conventional (culture, biochemical assay), molecular (rDNA sequencing), and/or reference laboratories were used to confirm and speciate the *Nocardia* isolates. Antibiotic susceptibility was determined using a combination of Etests (BioMerieux, Raleigh, NC), commercial laboratories, and the Sensititre Rapmyco microdilution panel (Thermo Fisher Scientific, Waltham, MA). Minimal inhibitory concentrations (MIC) interpretive standards for susceptible and resistant strains were in accordance with manufacturers and Clinical Laboratory Standards Institute (CLSI, Wayne, PA) guidelines [8]. Nonsusceptibility included both intermediate and resistant isolates.

## Results

**Fig. 1:**
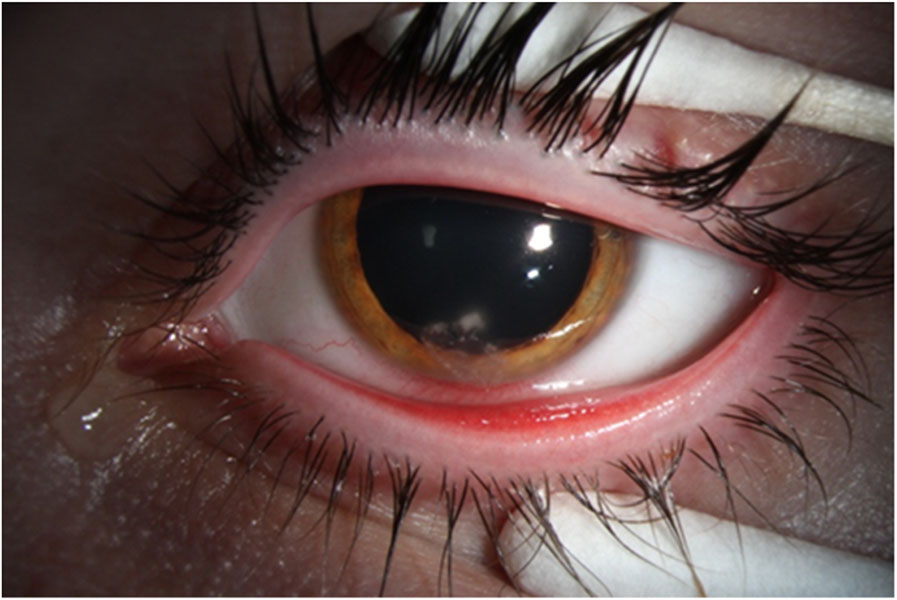
Patient with a *Nocardia* amikacinitolerans infiltrate. Classically described wreath-like, patchy lesions of *Nocardia* keratitis at six o’clock in a contact lens wearer

The current study includes 26 eyes of 26 patients. Overall, the median age was 32.5 years (*n* = 26; range: 16–66 years) and included 17 male and 9 female patients. A diverse group (*n* = 13) of *Nocardia* species were recovered among this patient population. *N. amikacinitolerans* (Fig. 1) was the most frequent isolate (*n* = 11, 42.3%) followed by *N. beijingensis* (*n* = 3, 11.5%), *N. arthritidis* (*n* = 2, 7.7%), and one each (3.8%) of remaining 10 isolates detailed in Table 1. Patients with *N. amikacinitolerans* keratitis were younger with a median age of 24 years (*n* = 11; range: 16–56 years).

**Table 1:** Clinical Characteristics of *Nocardia* Keratitis Cases (2014–2021)

Case	Age/Sex	Risk Factors	Days to Presentation	Presenting Visual Acuity	Presentation	Days to Resolution	Last Follow-up Visual Acuity	Species	Topical Steroid Use
1	49/F	Contact lens wear	5	20/20	1.4 mm corneal epithelial defect w/ underlying infiltrate	19	20/20	*Nocardia beijingensis/ pneumoniae/araoensis*	No
2	24/F	Contact lens wear; trauma	14	20/25	Inferior patchy infiltrate 5 mm × 2.5 mm with patchy overlying corneal epithelial defects	56	20/20	*Nocardia amikacinitolerans*	No
3	64/M	Contact lens wear (BCL)	14	No Light Perception	Central 1.6 mm × 1.6 mm white chalky infiltrate	Lost to Follow Up	No Light Perception	*Nocardia testacea*	Yes
4	20/F	Contact lens wear	19	20/70	2.2 mm × 2.2 mm inferonasal round patch of irregular multifocal white infiltrates, minimal corneal thinning, irregular overlying punctate corneal staining, no dendrites	54	20/25	*Nocardia beijingensis*	No
5	25/M	Contact lens wear	10	20/40–2	1 mm × 1.4 mm white fluffy opacity with irregular borders; satellite sub 1 mm round lesion, no staining, no neovascularization	44	20/70	*Nocardia amikacinitolerans*	No
6	21/M	Contact lens wear	14	20/30	Irregular epithelium with pseudodendritic appearance 5 mm non-continuously, scattered anterior stromal infiltrate with pannus	39	20/40–1	*Nocardia amikacinitolerans*	Yes
7	66/M	Trauma	7	0.5/200	2 mm × 2 mm corneal ulcer with corneal epithelial defect and Descemet’s folds	20	0.5/200	*Nocardia harenae*	Yes
8	55/M	Contact lens wear (soft); trauma	10	20/25	Inferior soupy 1.5 mm circular ulcer	Lost to Follow Up	Lost to Follow Up	*Nocardia amikacinitolerans*	No
9	56/F	Contact lens wear	5	20/70	7 discrete, round corneal epithelial defects with anterior stromal infiltrate underlying	6	20/40	*Nocardia amikacinitolerans*	No
10	19/M	Trauma	3	20/20	~ 0.9 mm × 0.9 mm patchy infiltrate, edges are more opaque and not contiguous, focal mild Descemet’s folds, surrounding infiltrate looks dense	49	20/20	*Nocardia farcinica*	No
11	24/M	Contact lens wear	70	20/30	2+ infiltrate, 4.5 mm × 2 mm ring infiltrate, no hypopyon	45	20/30	*Nocardia beijingensis/ pneumoniae*	No
12	38/M	Contact lens wear (soft)	13	20/400	Corneal epithelial defect 2 mm × 3 mm, questionable infiltrate on borders, no satellite (suspected HSV keratitis), corneal epithelial defect with neovascular limbal vessels. Central haze and infiltrate, mild punctate stain	190	20/30	*Nocardia arthritidis*	Yes
13	16/M	Contact lens wear	14	20/30	2.5 mm clusters of superficial infiltrates without thinning	43	20/25	*Nocardia amikacinitolerans*	Yes
14	52/M	Contact lens wear	30	20/20	Ring infiltrate with central haze	64	20/20	*Nocardia veterana*	Yes
15	30/M	Contact lens wear	10	20/25	Large lesion with raised edges and scattered staining	47	20/20	*Nocardia amikacinitolerans*	No
16	21/M	Contact lens wear	17	20/30	4.6 mm × 4.4 mm area of stromal infiltrate with discrete white opacities, white ring at the border 360 that stains, otherwise no staining. Some enlarged corneal nerves. No endothelial plaque	137	20/25	*Nocardia amikacinitolerans*	Yes
17	17/M	Contact lens wear (soft)	5	20/30	1 mm ×1 mm ulcer with small infiltrate at margin of lesion and overlying epithelial defect	9	20/20	*Nocardia amikacinitolerans*	No
18	52/F	Contact lens wear	4	20/20	Inferotemporal, inferior and superior pinpoint infiltrates, very pinpoint corneal epithelial defect	50	20/20	*Nocardia arthritidis*	Yes
19	36/F	Contact lens wear	10	20/25 + 1	Central corneal ulcer, 1.5 mm × 1.6 mm, with 25% thinning, underlying Descemet’s folds, diffuse epithelial edema, peripheral staining of ulcer edges	Lost to Follow Up	20/25–2	*Nocardia amikacinitolerans/ beijingensis*	Yes
20	35/M	Contact lens wear	9	20/400	Pannus, multiple small infiltrates 0.2 mm × 0.2 mm with overlaying corneal epithelial defect arranged roughly in a circle (nonconfluent), no dendrites	134	20/100	*Nocardia asteroides* complex	No
21	27/M	Contact lens wear	14	20/100	1 mm × 1 mm with infiltrate	7	20/80	*Nocardia endophytica*	Yes
22	16/M	Contact lens wear	Not Available	20/300	2.3 mm × 2.4 mm superficial corneal lesion, with pseudo dendrites emanating from the center of the lesion	49	20/20	*Nocardia beijingensis*	Yes
23	23/F	Contact lens wear	7	20/25	2.9 mm × 2.1 mm infiltrate concentrated on periphery of lesion. Scattered small corneal epithelial defects around periphery of lesion. Minimal cornea edema surrounding.	25	20/40	*Nocardia amikacinitolerans*	No
24	56/M	Trauma	7	20/40	Dendriform corneal epithelial defect with underlying opacity outside visual axis < 1 mm in size	16	20/50	*Nocardia amikacinitolerans*	No
25	38/F	Contact lens wear	7	20/30–2	1.8 mm epithelial defect with anterior stromal infiltrate at edges and surrounding haze. No thinning	3	20/40	*Nocardia bhagyanarayanae*	No
26	54/M	Contact lens wear	7	20/70	Not available	51	20/40	*Nocardia beijingensis*	Yes

Complete susceptibility data is summarized in Table 2; in vitro susceptibility daya was not available for a total of one isolate. Amikacin nonsusceptibility was determined in 64% of isolates (*n* = 16/25). All 11 of the *N. amikacinitolerans* isolates were resistant to amikacin and constituted 73.3% (*n* = 11/15) of the amikacin nonsusceptible isolates documented during the study. Of note, 100% of isolates were susceptible to either trimethoprim sulfamethoxazole or linezolid.

**Table 2:** In vitro antibiotic susceptibility profiles of *Nocardia* keratitis isolates

*Nocardia* Species	number of isolates	Amikacin	Tobramycin	Ciprofloxacin	Moxifloxacin	Clarithromycin	Doxycycline	Minocycline	Trimethoprim-Sulfa	Linezolid	Imipenem	Amoxicillin-Clavulanic Acid	Ceftriaxone	Cefepime
*Nocardia amikacinitolerans*	11	0%	82%	0%	9%	0%	36%	100%	100%	100%	18%	100%	27%	0%
*Nocardia beijingensis*	3	33%	100%	0%	0%	0%	33%	100%	100%	100%	0%	100%	67%	67%
*Nocardia arthritidis*	2	100%	50%	33%	50%	50%	50%	50%	100%	100%	0%	50%	50%	50%
*Nocardia harenae*	1	100%	100%	100%	100%	100%	100%	100%	100%	100%	100%	100%	100%	100%
*Nocardia farcinica*	1	100%	0%	0%	100%	0%	0%	0%	100%	100%	100%	100%	0%	0%
*Nocardia veterana*	1	100%	0%	0%	0%	100%	0%	0%	100%	100%	100%	0%	0%	100%
*N. asteroides complex*	1	0%	100%	0%	0%	0%	0%	0%	100%	100%	0%	0%	0%	0%
*N. endophytica*	1	100%	100%	100%	100%	100%	100%	100%	100%	100%	0%	0%	100%	100%
*Nocardia bhagyanarayanae*	1	100%	100%	0%	0%	0%	0%	100%	100%	100%	0%	100%	0%	100%
*Nocardia testacea*	1	0%	100%	0%	0%	100%	100%	100%	100%	100%	0%	100%	100%	0%
*N. beijingensis/pneumoniae*	1	100%	100%	100%	100%	0%	0%	100%	100%	100%	100%	0%	100%	100%
*Nocardia beijingensis/pneumoiae/avagensis*	1	0%	100%	0%	0%	0%	0%	0%	100%	100%	100%	100%	100%	0%
% Susceptible		36%	80%	16%	24%	20%	36%	80%	100%	100%	28%	80%	44%	32%
% Resistant		64%	12%	80%	60%	80%	8%	0%	0%	0%	44%	12%	24%	60%
% Intermediate		0%	8%	4%	16%	0%	56%	20%	0%	0%	28%	8%	32%	8%
MIC50 (ug/ml)		< 32 (R)	< 1 (S)	> 4 (R)	> 4 (R)	> 16 (R)	< 2 (I)	< 1 (S)	< 0.5 (S)	< 2 (S)	> 8 (R)	< 4 (S)	16 (I)	> 32 (R)
MIC90 (ug/ml)		> 64 (R)	> 12.8(R)	> 4 (R)	> 8 (R)	> 16 (R)	> 6.4(R)	3.2 (I)	3.9 (S)	3.2 (S)	> 51.2(R)	> 25.6(R)	> 64 (R	> 32 (R)

Mean presenting BCVA (*n* = 25) was 20/60 ± 2.3 lines. (Table 1). At presentation, 64% (*n* = 16/25) of the patients had a BCVA of 20/40 or better and a median time from symptom onset to presentation of 10 days (*n* = 25; range: 3–70 days). The mean post-treatment BCVA (*n* = 21) was 20/40 ± 2.7 lines with a median treatment duration of 44 days (*n* = 23; range: 3–190 days). A final post-treatment BCVA of 20/40 or better was achieved in 71.4% (*n* = 15/21) of patients. Overall, there was no significant difference in presenting versus last follow-up BCVA.

Contact lens wear was the leading identified risk factor for *Nocardia* keratitis among the study population (Table 1). A history of contact lens wear was present in88.5% (*n* = 23/26) of patients; the remaining non-contact-lens cases were either associated with trauma. Trauma-related *Nocardia* keratitis was documented in 15.4% (*n* = 4/26) of total cases. South Florida patients presenting with *Nocardia* keratitis were six times (23:4) more likely to be associated with contact lens wear than with trauma.

## Discussion

The current study is the largest series to date on risk factors and amikacin-resistance among patient with *Nocardia* keratitis in the United States. The current series differs compared to reports from Asia by species diversity, risk factors, and amikacin susceptibility profiles [5, 7]. Compared to the largest reported series from India (*n* = 116) [5], patients in this current series were younger, presented earlier, had better presenting/final BCVA, and healed faster.

The true prevalence of *Nocardia* keratitis in the United States is unknown, but prior to this study, only one series of three or more patients with *Nocardia* keratitis had been reported in the United States [9]. Hirst reported on a series of eight patients in 1979. Since then, only sporadic cases (*n* = 17) have been reported from 10 states and Washington DC [9–25]. Overall, 72% (*n* = 18/25) have been reported from northern states with only six reported from southern states including Florida (*n* = 4), Georgia (*n* = 1), and Texas (*n* = 1). Nine of the 17 (52.9%) reports have been contact-lens associated supporting the evolving epidemiology in other parts of the United States.

*Nocardia amikacinitolerans* was the predominant identified *Nocardia* species among South Florida isolates in the current study resulting in keratitis; this is the second reported case series worldwide. Among more than 200 *Nocardia* keratitis cases reported from South India in the last three decades, none have been identified as *N. amikacinitolerans* [4, 5, 7].

Amikacin nonsusceptibility was found to be 64% in this case series. DeCroos and colleagues reported a resistance rate of 3% for their 116 *Nocardia* keratitis isolates over an 11-year period [5]. Sporadic, but increasing amikacin resistance have been reported for a diverse group of *Nocardia* keratitis isolates including *N. tranvalensis* [26], and members of the *N. asteroides* complex [24].

In vitro susceptibilities for *Nocardia* species are strain specific. It is important to run in vitro susceptibility testing to determine the most effective drugs for ocular *Nocardia* infections [1, 5, 27, 28]. Based on in vitro data in this current study, trimethoprim sulfamethoxazole and linezolid demonstrated 100% susceptibility rates. Given its wider availability, trimethoprim sulfamethoxazole may be the preferred antibiotic agent in treating *Nocardia* keratitis and specifically, amikacin-resistant cases of *Nocardia* keratitis. Data from the Steroids for Corneal Ulcer Trial (SCUT) study confirmed the correlation between increasing drug minimal inhibitory concentrations and patient’s outcomes.

Contact lens use was the leading risk factors identified among South Florida Nocardia keratitis patients. Contact lens wear was not a recognized risk factors among the 116 cases reported by DeCroos and colleagues nor among the 55 patients in the SCUT study [4]. However, contact lens associated *Nocardia* keratitis may be increasing worldwide and in the United States [28–30]. This infection should be considered with a higher index of suspicion in contact lens wearers with refractory corneal ulcers. Specific details regarding contact lens type or specific hygiene regimen surrounding contact lens use were unable to be determined in this study.

## Conclusion

*Nocardia* keratitis is rare and its clinical presentation is diverse. Contact lens wear is the leading risk factor of *Nocardia* keratitis in South Florida and has been the most commonly associated risk factor in the United States for the last 10 years. First line therapy with amikacin alone may lead to clinical failure consider trimethoprim sulfamethoxazole. Early collaboration with a microbiology laboratory to speciate and perform susceptibility testing can lead to favorable visual outcomes.

## Data Availability

The datasets used and analyzed during this study are available from the corresponding author on reasonable request.

## Abbreviations

IRB: Institutional Review Board
BCVA: Best Corrected Visual Acuity
MIC: Minimal inhibitory concentration
SCUT: Steroids for Corneal Ulcer Trial
